# Rituximab in patients with rheumatoid arthritis in routine practice (GERINIS): six-year results from a prospective, multicentre, non-interventional study in 2,484 patients

**DOI:** 10.1186/ar4521

**Published:** 2014-03-26

**Authors:** Joerg Wendler, Gerd R Burmester, Helmut Sörensen, Andreas Krause, Constanze Richter, Hans-Peter Tony, Andrea Rubbert-Roth, Peter Bartz-Bazzanella, Siegfried Wassenberg, Iris Haug-Rost, Thomas Dörner

**Affiliations:** 1 Rheumatologische Schwerpunktpraxis, Moehrendorfer Strasse 1c, D-91056 Erlangen, Germany; 2 Department of Rheumatology and Clinical Immunology, Charité – University Medicine Berlin, Free University and Humboldt University Berlin, Schumannstr 20-21, 10098 Berlin, Germany; 3 Rheumaprojekt Berlin-Brandenburg, Argentinische Allee 42, 14163 Berlin, Germany; 4 Immanuel Krankenhaus, Lindenberger Weg 19, D-13125 Berlin, Germany; 5 Rheumatology Medical Centre, Stuttgart, Germany; 6 Medizinische Klinik und Poliklinik II, Rheumatology/Clinical Immunology, Klinikstr 6-8, 97070 Würzburg, Germany; 7 Medical Clinic I, University of Cologne, Josef-Stelzmann-Str 9, 50931 Cologne, Germany; 8 Klinik für Internistische Rheumatologie, Mauerfeldchen 25, 52146 Würselen, Germany; 9 Ev. Fachkrankenhaus Ratingen, Rosenstraße 2, D-40882 Ratingen, Germany; 10 Department of Rheumatology, Specialty Care/Medical Affairs Roche Pharma AG, Emil-Barell-Straße 1, D-79639 Grenzach-Wyhlen, Germany; 11 CC12, Department of Medicine Rheumatology and Clinical Immunology and DRFZ Berlin, Schumannstr 20/21, 10098 Berlin, Germany

## Abstract

**Introduction:**

The aim of this study was to evaluate the safety and efficacy of rituximab (RTX) in a large cohort of patients with rheumatoid arthritis in routine care, and to monitor changes in daily practice since the introduction of RTX therapy.

**Methods:**

This was a multicentre, prospective, non-interventional study conducted under routine practice conditions in Germany. Efficacy was evaluated using Disease Activity Score in 28 joints (DAS28) and Health Assessment Questionnaire-Disability Index (HAQ-DI). Safety was assessed by recording adverse drug reactions (ADRs). Physician and patient global efficacy and tolerability assessments were also evaluated.

**Results:**

Overall, 2,484 patients (76.7% female, mean age 56.4 years, mean disease duration 11.7 years) received RTX treatment (22.7% monotherapy). The total observation period was approximately six-years (median follow-up 14.7 months). RTX treatment led to improvements in DAS28 and HAQ-DI that were sustained over multiple courses. DAS28 improvements positively correlated with higher rheumatoid factor levels up to 50 IU/ml. Response and tolerability were rated good/very good by the majority of physicians and patients. Mean treatment intervals were 10.5 and 6.8 months for the first and last 400 enrolled patients, respectively. Infections were the most frequently reported ADRs (9.1%; 11.39/100 patient-years); approximately 1% of patients per course discontinued therapy due to ADRs.

**Conclusions:**

Prolonged RTX treatment in routine care is associated with good efficacy and tolerability, as measured by conventional parameters and by physicians’ and patients’ global assessments. Rheumatoid factor status served as a distinct and quantitative biomarker of RTX responsiveness. With growing experience, physicians repeated treatments earlier in patients with less severe disease activity.

## Introduction

The anti-CD20 monoclonal antibody rituximab (RTX) was licensed in 2006 in combination with methotrexate (MTX) for the treatment of severe, active rheumatoid arthritis (RA) in adult patients with an inadequate response to disease-modifying antirheumatic drugs (DMARDs) including one or more tumour necrosis factor (TNF) inhibitors. Based on the pioneering idea that RTX might be of value in the treatment of seropositive RA, a proof of concept study confirmed its efficacy and safety in combination with MTX and thereby provided strong evidence for the role of B cells in this disease [1]. RTX is distinct from other biological DMARDs, with regards to its mode of action which involves the targeting of CD20+ B cells resulting in the inhibition of B-cell-mediated inflammatory responses. Another unique feature of RTX is the long interval between treatment courses; the selective depletion of CD20-positive B cells by RTX results in a long duration of therapeutic response with each course of treatment [2]. RTX retreatment is generally recommended at around six months based on clinical evaluation [3], whereas other RA biologicals are administered using monthly or more frequent regimens. The less frequent dosing schedule of RTX means that more prolonged follow-up may be needed in order to properly evaluate physicians’ and patients’ experiences with this therapy.

Extensive data on the long-term efficacy and safety profile of RTX are now available, mainly from long-term follow-up of patients participating in the RTX clinical trial programme. Five-year efficacy data from the REFLEX trial extension have recently been reported [4], as have been safety data from a pooled analysis of all RTX clinical trials with a follow-up of 10 years, involving up to 17 courses [5]. However, clinical trials are biased by the requirements of patient exclusion and inclusion criteria, and it is estimated that only about 30% of daily practice patients would be eligible for such studies [6]. Consequently, data obtained in real-life settings are also valuable. Such data from RTX-treated patients have been reported from a number of European registries, although generally involving relatively shorter periods of follow-up [7-11].

This very large, non-interventional study was initiated in Germany in 2006 when RTX was first authorised for RA treatment. The main purpose of the study was to evaluate the safety and efficacy of RTX in routine RA care. An additional objective was to monitor changes in daily practice during the period following the introduction of RTX, for example, with regard to retreatment or concomitant therapy, and whether specific variables, such as patient age, influence treatment outcomes.

## Materials and methods

### Study design

This was a multicentre, prospective, non-interventional study, the primary objective of which was to assess the long-term efficacy and safety of RTX in patients with active RA in a routine practice setting. Participating physicians were rheumatologists practising at 215 outpatient clinics or private practices in Germany. A list of study investigators and sites is provided in Additional file 1. Patients received RTX treatment and retreatment according to the discretion of the physician. Patient data were collected for a period of two years after the start of the first RTX treatment course. If patients required retreatment, the two-year observation period was restarted at the point of RTX retreatment. Clinical visits were documented at baseline (first infusion), Day 15 (second infusion) and, as available, at months 4, 8, 12, 16, 20 and 24.

In accordance with Section 67, Sub-Section 6 of the German Drug Law, the Federal Panel Doctors' Association, the Central Federal Association of health insurance funds and the competent higher federal authority were notified regarding the conduct of this non-interventional study. Ethical approval of the study and patient written consent were not obtained as neither was mandatory in Germany for non-interventional studies when the study commenced in 2006. The study was conducted in accordance with the principles of the Declaration of Helsinki.

### Assessments

The main parameter for evaluating efficacy was the Disease Activity Score in 28 joints (DAS28). Assessment of functional disability was performed using the Health Assessment Questionnaire-Disability Index (HAQ-DI). Safety was assessed by the occurrence of adverse drug reactions (ADRs) and infusion reactions (IRs). Measurement of rheumatoid factor (RF) was performed at the individual study sites, using nephelometry which primarily detects IgM RF. All clinical assessments were made by fully trained qualified health care professionals, although we accept that some degree of variation in both the competence and techniques applied across >200 outpatient clinics and private practices is likely to have occurred and may have influenced the results to some extent. Additionally, physicians and patients were asked to assess the global efficacy and tolerability of RTX therapy using the following four categories: very good, good, moderate and poor.

### Statistical analyses

The safety population comprised all patients who had received at least one RTX infusion. The efficacy population comprised all patients with a primary diagnosis of RA and in whom at least one post-baseline efficacy measurement had been documented. All efficacy and safety parameters were analysed descriptively using SAS version 8.2 software (SAS Institute, Cary, North Carolina, USA). For numerical data, mean, standard deviation, median, range and interquartile ranges were calculated. Categorical data were analysed by calculating absolute and relative frequencies. Missing values were not imputed. Changes from baseline in efficacy parameters were used as outcome variables. Subgroup analyses were performed based on the following parameters: time of inclusion (first 400 enrolled patients versus last 400 enrolled patients), RF level, age and sex. Due to decreasing patient numbers with each treatment course, the efficacy evaluations in this analysis focused on the first three treatment courses. Correlation between baseline RF level and change in DAS28 was analysed using the Locally Estimated Scatterplot Smoothing (LOESS) non-parametric regression method [12]. In brief, this involves the selection of a specific span (bandwidth) of points along the x-axis adjacent to the point being predicted. A regression equation is then fitted for the selected points through this subset of data giving more weight to points closest to the value being predicted. The resulting equation is then used to predict the value for the selected point. The data are then shifted one point to the right and the process is repeated. The resulting predicted values and confidence limits are connected together with lines. The smaller the bandwidth, the less smooth the final line. To improve the comparability of the data, the LOESS analysis included the subgroup of seropositive and seronegative patients with RF levels determined using the most frequently used diagnostic tests (normal ranges of <14, 15 or 20 IU/ml).

## Results

Data from 2,484 patients including all treatment courses over a period of approximately six years (median follow-up 14.7 months, range 0.0 to 64.8 months) were collected. The safety population comprised 2,484 patients, while the efficacy population comprised 2,424 patients. Consistent and evaluable efficacy data for at least three RTX treatment courses were available for a subgroup of 902 patients (the evaluable efficacy population). Most patients (98.1%) were treated using RTX 2 × 1,000 mg per course. Times to retreatment changed during the course of the study, and with each additional course. In the efficacy population, the mean retreatment interval was 9.6 months between courses 1 and 2, and 7.7 months between courses 5 and 6. Demographics and baseline characteristics are summarised in Table 1.

**Table 1:** Demographics and baseline characteristics

	**Safety population (*n =*** 2,484^*^)	**Patients enrolled early (*n =*** **400)**	**Patients enrolled late (*n =*** **400)**
Female, *n* (%)	1906 (76.7)	302 (75.5)	326 (81.5)
Age (years), mean ± SD	56.4 ± 12.4 (*n =* 2,474)	55.1 ± 12.1	56.9 ± 12.4
Duration of disease before study, years, mean ± SD	11.7 ± 9.6 (*n =* 2,168)	12.3 ± 9.3	11.9 ± 9.9
RF-positive, *n* (%)	1477 (77.3) (*n =* 1,910)	277 (78.5) (*n =* 353)	232 (77.6) (*n =* 299)
Treatment duration (months) over all study courses, mean ± SD	17.2 ± 13.7 (*n =* 2,484)	22.4 ± 17.0	9.1 ± 7.9
No. of previous DMARD baseline treatments including TNF antagonists, median	4	5	4
No. of previous conventional DMARD baseline treatments, median	3	3	2
No. of baseline TNF antagonists, median^†^	1	2	1
Previous TNF antagonists, *n* (%)^†^			
0	510 (20.5)	66 (16.5)	89 (22.3)
1	827 (33.3)	93 (23.3)	163 (40.8)
2	785 (31.6)	135 (33.8)	122 (30.5)
3	362 (14.6)	106 (26.5)	26 (6.5)
No. of different TNF antagonists per patient, mean ± SD	1.4 ± 1.0	1.7 (1.0)	1.2 (0.9)
Most frequent previous			
DMARD baseline treatments, *n* (%)			
Leflunomide	1690 (68.0)	285 (71.3)	256 (64.0)
Methotrexate	1661 (66.9)	291 (72.8)	276 (69.0)
Adalimumab	1385 (55.8)	245 (61.3)	200 (50.0)
Etanercept	1360 (54.8)	265 (66.3)	205 (51.3)
Sulphasalazine	1145 (46.1)	215 (53.8)	168 (42.0)
Infliximab	736 (29.6)	171 (42.8)	80 (20.0)
Baseline DAS28, mean ± SD^‡^	5.7 ± 1.2 (*n =* 1,954)	5.9 ± 1.2 (*n =* 384)	5.4 ± 1.3 (*n =* 349)
Baseline HAQ, mean ± SD^‡^	1.6 ± 0.7 (*n =* 2,078)	1.74 ± 0.70 (*n =* 389)	1.56 ± 0.73 (*n =* 384)
Time between first and second course (months), mean ± SD^‡^	9.6 ± 4.4 (*n =* 1,306)	10.5 ± 4.9 (*n =* 250)	6.8 ± 1.6 (*n =* 143)

^*^Missing values were not imputed. Total patient number was 2,484, unless stated. ^†^For course 1.

^‡^Efficacy population.

DAS28, Disease Activity Score in 28 joints; DMARD, disease-modifying antirheumatic drug; HAQ, Health Assessment Questionnaire; RF, rheumatoid factor; SD, standard deviation; TNF, tumour necrosis factor-alpha.

### Efficacy

In the efficacy subgroup, baseline DAS28 scores at courses 1, 2 and 3 were 5.7, 5.0 and 4.6, respectively. Mean change from baseline in DAS28 during the first treatment course was -1.4 points at month 4 and -1.4 points at month 8. After three treatment courses, mean DAS28 decreased to 3.6 at both the four-month and eight-month time points. In parallel with the improvements in DAS28 score, an increasing proportion of patients achieved low disease activity (LDA) status with each additional treatment course. The proportion of patients with LDA increased from 3.5% at baseline prior to course 1 to 23.7% (course 1, month 8), 30.8% (course 2, month 8) and 42.4% (course 3, month 8). Patients with exposure to more than one previous TNF inhibitor also benefitted from RTX retreatment: the proportion of patients with DAS28 remission increased from 9.8% (course 1, month 8) to 29.5% (course 3, month 8).

Physician-reported mean swollen joint count (28 joints) decreased from 7.9 at baseline to 4.1 at month 4, and to 4.1 at month 8 of course 1, with further decreases observed during each subsequent course. In parallel, tender joint count (28 joints) decreased from 11.0 at baseline to 6.1 at month 8 of course 1, and to 4.3 at month 8 of course 3.

During the first treatment course, mean HAQ-DI values showed clinically meaningful improvements (a decrease of >0.22 points) of 0.22 points between baseline and month 4 and of 0.24 points between baseline and month 8. High proportions of patients showed a clinically meaningful decrease of HAQ-DI at month 8 following each RTX course even though the baseline values were lower: course 1, 48.6%; course 2, 42.0%; course 3, 32.6%.

### Subgroup analyses

**Figure 1:**
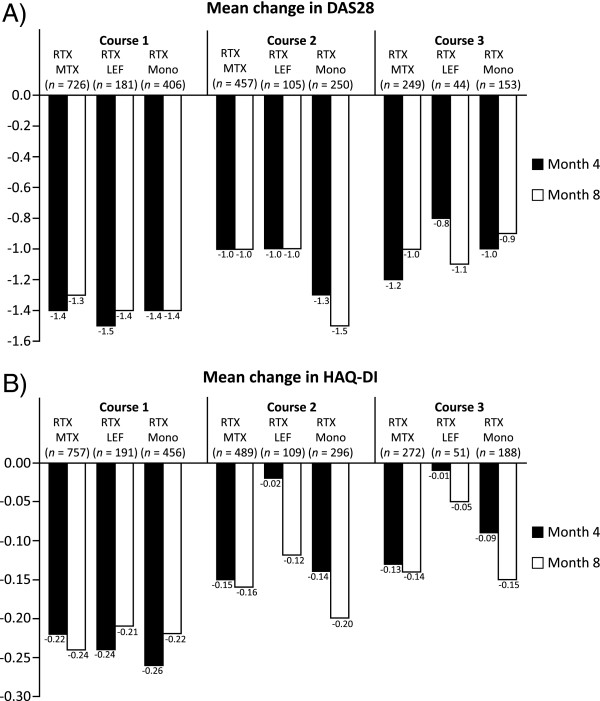
**Efficacy of rituximab as monotherapy and in combination with methotrexate and leflunomide. A)** DAS28 and **B)** HAQ-DI. The changes are with reference to the baseline values at each course. DAS28: Disease Activity Score in 28 joints, HAQ-DI: Health Assessment Questionnaire-Disability Index, LEF: leflunomide, MTX: methotrexate, RTX: rituximab.

A total of 564 patients (22.7%) received RTX as monotherapy. This subgroup was comparable to the total population with regard to demographic and baseline characteristics (76.4% female; mean age 59.3 years; mean disease duration 12.6 years; 79.2% RF-positive; and a median of four (mean 4.6) previous conventional DMARD therapies and a median of one (mean 1.4) previous TNF inhibitor). The efficacy of RTX monotherapy was comparable to that of RTX combined with MTX (*n =* 879) and leflunomide (LEF) (*n =* 215), with similar results for the investigated parameters, including DAS28, HAQ-DI (Figure 1) and time intervals between treatments.

Efficacy outcomes were also analysed according to patient age group (Table 2). Changes in DAS28 during courses 1 and 2 were similar in age groups <40 years, 40 to <60 years and ≥60 years.

**Table 2:** Overview of safety and efficacy of RTX treatment stratified by age

	**All patients**	**Age group (years)**		
		**<40**	**40 to <60**	**≥60**
**Safety, n (%)**				
No. of patients	2,484	204	1,187	1,029
ADRs	532 (21.4)	52 (25.5)	264 (22.2)	200 (19.4)
Serious ADRs	76 (3.1)	5 (2.5)	30 (2.5)	33 (3.2)
Infusion reactions	157 (6.3)	25 (12.3)^*^	83 (7.0)^*^	42 (4.1)^*^
Deaths	9 (0.4)	0	3 (0.3)	6 (0.6)
**Efficacy, DAS28 (mean [SD])**				
Course 1				
No. of patients (baseline)	1,954	163	975	814
Baseline	5.7 (1.2)	5.3 (1.3)	5.7 (1.2)	5.8 (1.2)
Month 4	4.3 (1.4)	4.0 (1.5)	4.3 (1.4)	4.3 (1.3)
Month 8	4.3 (1.4)	3.8 (1.7)	4.4 (1.5)	4.3 (1.3)
Course 2				
No. of patients (baseline)	1,315	117	660	537
Baseline	5.0 (1.3)	4.7 (1.5)	5.0 (1.3)	5.1 (1.2)
Month 4	4.0 (1.3)	3.6 (1.4)	4.0 (1.4)	4.0 (1.3)
Month 8	4.0 (1.4)	3.5 (1.6)	4.1 (1.3)	3.9 (1.4)

^*^Significantly different between age groups; *P* <0.005 (Fisher’s exact test). ADR, adverse drug reaction; DAS28, Disease Activity Score in 28 joints; RTX, rituximab; SD, standard deviation.

The first 400 patients entered the study between July 2006 and February 2007, while the last 400 patients were enrolled between January and October 2009. The interval between these two periods was shorter than initially planned due to faster than expected enrolment. In general, physicians tended to include patients with less severe disease later in the study; baseline DAS28 was higher in the early group compared with the later group (mean 5.9 versus 5.4). In addition, patients in the early group had received a greater median number of DMARDs at baseline (5.1 versus 3.8) and were on average slightly younger than those in the later group (55.1 versus 56.9 years). The mean time to retreatment was considerably longer in the early group compared with the later group (10.5 versus 6.8 months) (Table 1).

**Figure 2:**
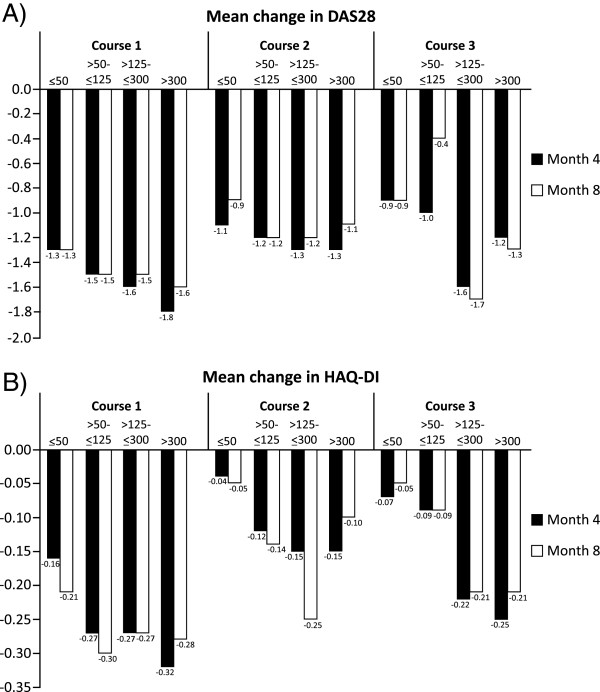
**Changes from baseline in DAS28 and HAQ-DI according to baseline rheumatoid factor level. A)** DAS28 and **B)** HAQ-DI. DAS28: Disease Activity Score in 28 joints, HAQ-DI: Health Assessment Questionnaire-Disability Index.

In order to assess the influence of serological status on responsiveness to RTX, the efficacy population was stratified according to RF levels at baseline. Analysis across these subgroups revealed that both DAS28 and HAQ-DI tended to show greater reductions over four and eight months in patients with higher RF levels at baseline (Figure 2). This trend was observed for all treatment courses.

**Figure 3:**
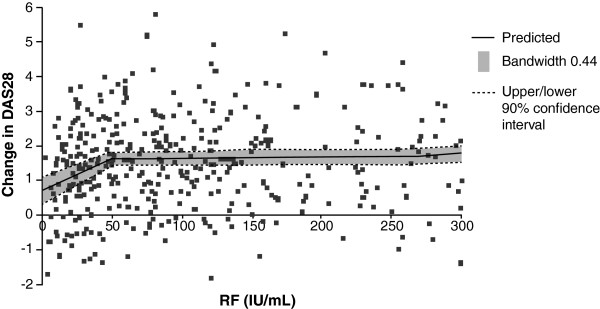
**LOESS analysis of the relation between RF level and DAS28.** Each data point indicates change in DAS28 from baseline at month 4. DAS28: Disease Activity Score in 28 joints, RF: rheumatoid factor.

Based on the data obtained by LOESS analysis, a direct correlation between baseline RF levels and responsiveness to RTX was found (spearman correlation coefficient 0.21; Figure 3). Responsiveness to RTX, in terms of DAS28 change from baseline at four months, improved in association with increased RF level up to approximately 50 IU/ml. Baseline RF level above 50 IU/ml was not associated with any further increase in RTX responsiveness. The LOESS analysis confirmed that RF-positive patients respond better to RTX.

### Global assessment of efficacy and tolerability

**Figure 4:**
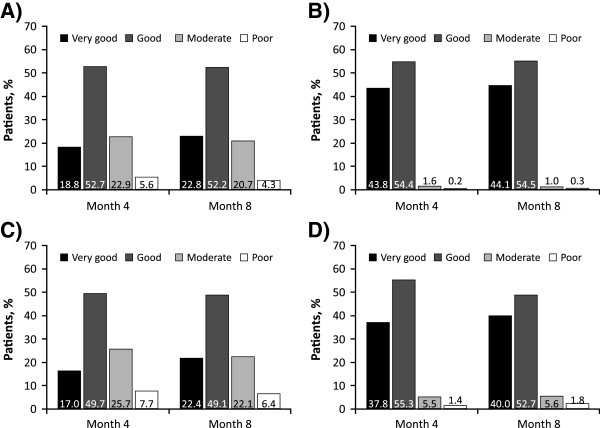
**Global ratings of efficacy and tolerability by physicians and patients during course 1. A)** Efficacy, physician. **B)** Tolerability, physician. **C)** Efficacy, patient. **D)** Tolerability, patient.

The efficacy and tolerability of RTX treatment were rated to be good or very good by the majority of patients and physicians (Figure 4). At month 8 of course 1, 75.0% of physicians and 71.5% of patients considered the efficacy of RTX to be good or very good, while 98.6% of physicians and 92.7% of patients rated the tolerability of RTX to be good or very good. Ratings tended to improve during subsequent courses. In spite of the high overall percentage of good to very good ratings, corresponding to a generally good acceptance, patients tended to give slightly lower ratings than physicians.

### Safety

ADRs were reported in 532 patients (21.4%; 39.58/100 patient-years) overall (Table 2). The most frequent ADRs were infections and parasitic diseases (9.1%; 11.39/100 patient-years), with the most frequent infection being nasopharyngitis (1.8%; 1.61/100 patient-years). Skin and subcutaneous tissue disorders were also common (5.5%; 5.48/100 patient-years), and mainly pruritus (1.5%; 1.39/100 patient-years). All other reported ADRs occurred at a frequency of <5% (<5/100 patient-years). Serious ADRs were reported by 76 patients (3.1%; 4.44/100 patient-years). Overall, IRs were reported in 157 patients (6.3%; 3.62/100 patient-years). The incidence of IRs was highest during course 1 (4.3% (95/2,195)) and then declined with subsequent courses (3.0% (46/1,509)) for course 2 and 1.8% [16/909] for course 3. RTX infusions were interrupted due to ADRs in 30 patients. Nine patients (0.4%; 0.40/100 patient-years) had serious IRs.

Cases of sepsis were rare: general sepsis (n = 3), septic shock (n = 2), and urosepsis (n = 1). Single cases of the following fungal infections were reported (as described in the safety report): fungal infection, sinusitis due to fungal infestation, *tinea corporis*, *tinea pedis*, dermal fungal infection, gastointestinal candidosis, oral candidosis, vaginal candidosis and vulvovaginal fungal infection. No cases of hepatitis or atypical mycobacterial infection were reported. One patient died who had tuberculosis and sepsis. No cases of progressive multifocal leukoencephalopathy were reported throughout the observation period.

The proportions of patients reporting ADRs and serious ADRs were also similar in the three age groups. There were no apparent differences between RF-positive and RF-negative patients in the frequency of overall ADRs (23.1 versus 21.1%, respectively) or serious infections and parasitic diseases (1.2 versus 0.9%, respectively).

Nine patients died during the study. Six of these deaths were considered not related to RTX. In one case causality was assessed as not applicable and two cases were assessed as possibly related (one patient died due to postoperative complications including sepsis and the other due to an acute respiratory distress syndrome).

## Discussion

Non-interventional studies can provide valuable data with regard to the efficacy and safety of therapies in a real-life setting. This study, conducted in routine clinical practice in Germany over a period of approximately six years starting soon after the marketing authorisation of RTX, is possibly the largest of its type with the inclusion of over 2,400 patients. The results indicate that RTX treatment was associated with favourable efficacy outcomes over multiple courses, with significant decreases in DAS28 accompanied by clinically meaningful changes in HAQ-DI. The findings are in line with those previously reported from RTX clinical trials [4,13] and European registries [7,8,10]. In addition, the results provide convincing documentation of changes in clinical practice over time and the first evidence indicating a quantitative relationship between RF level and response to RTX. A sub-analysis of our study further demonstrated the effectiveness of RTX in older patients, with outcomes observed in patients over 60 years of age comparable with those seen in younger patients. Interestingly, IRs were less frequent in patients of 60 years or older compared with patients younger than 60 years old. To our knowledge this is the first report of the influence of age on RTX efficacy and safety.

A longitudinal analysis of the data suggests that there have been some changes in physicians’ practices since RTX was first licensed for treatment of RA in 2006. By comparing data from the first 400 enrolled patients with those from the last 400 enrolled patients, a period separated by approximately 2.5 years, the results suggest that, over time, physicians changed to using RTX in patients with less severe disease. In addition, RTX retreatment intervals generally shortened over the time of the study. These observations of changes in daily practice likely reflect the introduction into clinical practice in Germany of guidelines recommending a treatment to target approach for RA biological therapies [3,14,15]. It is worth noting that a small minority (approximately 3.5%) of patients were treated with rituximab despite having LDA; the reasons for this non-standard approach are not clear as this information was not collected during the study. However, a number of explanations are possible, including individual consideration of patient treatment according to disease history; discontinuation of prior therapy due to intolerability while having LDA; or physician preference to target remission even when LDA had been achieved.

The summary of product characteristics recommends that RTX is administered in combination with MTX [16]. It is interesting to note, therefore, that nearly one-quarter (23%) of patients in the current study received RTX as monotherapy. The reasons for this relatively high proportion are not clear. MTX may be contraindicated in some patients and not tolerated by others, although other reasons, such as physicians’ or patients’ preference, may also have been involved. The efficacy outcomes associated with RTX monotherapy were generally similar to those achieved with RTX in combination with MTX or LEF. Interestingly, the present analysis of the complete data set differs from a previous interim analysis of 995 patients, which indicated that RTX plus LEF provided superior outcomes (EULAR responses) to that of RTX plus MTX [17]. Other previous studies have reported that RTX monotherapy is similar to RTX plus MTX in terms of short-term efficacy [18,19], while another study reported that RTX plus LEF gave better outcomes than RTX alone or in combination with MTX [20]. Data from a phase II trial indicated that the probability of achieving higher hurdle clinical endpoints, such as ACR50 or ACR70, is increased when RTX is administered in combination with MTX rather than as monotherapy [1].

Patients seropositive for RF have been shown previously to exhibit greater efficacy responses to RTX than those who are seronegative [7,9,11,18,21,22]. In this non-interventional study, patients with higher RF levels tended to have greater DAS28 and HAQ responses than those with lower levels. These data further support the use of RF status as a biomarker for predicting RTX responsiveness and may extend this correlation to a quantitative relationship. The results of the LOESS analysis indicated that RTX responsiveness increases up to an RF level of approximately 50 IU/ml, with no further increases seen above this level. Although seronegative and low-level RF positive patients tended to respond less well to RTX, the results indicate that some of these patients, perhaps around 50%, may still benefit from RTX therapy.

The safety profile of RTX has been established primarily from long-term follow-up of patients in the RTX clinical trial programme [5,23]. Real-life studies, such as the current non-interventional study, are invaluable as they include patients with significant comorbidities or other demographic and disease characteristics that would exclude them from formal clinical trials. RTX was rated as good or very good in terms of both efficacy and tolerability by the majority of physicians and patients in this study. As would be expected from a real-life study, the proportion of patients reporting ADRs was generally lower than that reported in the RTX clinical trials [5,23].

The study has a number of possible limitations due to the non-interventional design with lack of a control group that limit a more reliable evaluation of efficacy and tolerability of RTX. Moreover, adverse event rates may be underestimated due to less stringent requirements when compared with randomised clinical trials. Finally, assessment of subgroups (for example, age) was somewhat limited by smaller patient numbers in these groups. Despite these limitations, the study provides insights into daily clinical practice beyond a rather selected patient group often enrolled in clinical studies.

## Conclusions

In conclusion, the results of this large non-interventional study involving 2,484 patients with observation over six years indicate that RTX is efficacious and well tolerated in routine care in Germany, including in older patients, with improved efficacy results in RF-positive patients. In addition, the results demonstrate that RTX monotherapy and in combination with LEF may represent an alternative for some patients intolerant to MTX. This study also provides evidence that changes in the use of RTX to treat RA have occurred in recent years, thus indicating that physicians have adopted this therapy and have developed how it is used in routine care.

## Abbreviations

ADR: Adverse drug reaction; DAS28: Disease activity score in 28 joints; DMARDs: Disease-modifying antirheumatic drugs; HAQ-DI: Health assessment questionnaire-disability Index; IRs: Infusion reactions; LDA: Low disease activity; LEF: Leflunomide; LOESS: Locally estimated scatterplot smoothing; MTX: Methotrexate; RA: Rheumatoid arthritis; RF: Rheumatoid factor; RTX: Rituximab; SD: Standard deviation; TNF: Tumour necrosis factor.

## Competing interests

JW has received research grants and consultancy fees from Roche/Chugai. G-RB has received honoraria from Roche for consulting and lectures and his institution has received research grants from Roche. AK reports personal fees from Roche, grants and personal fees from Abbott/AbbVie and Roche/Chugai, and personal fees from Pfizer, BMS, UCB, Janssen and MSD. HS and CR report no conflicts of interest. H-PT has attended speakers’ bureaus and has acted as a consultant for Abbvie, BMS, Chugai, MSD, Roche and UCB. AR-R has received honoraria for lectures and consultancies from Roche, Chugai, MSD, UCB, Pfizer, Abbvie and BMS. PB-B has received speaker honoraria from Roche, Chugai, Pfizer, Abbvie and BMS. SW reports personal fees and grants from Roche Pharma during the conduct of the study. IH-R is an employee of Roche Pharma AG Germany. TD reports grants and personal fees from Roche/Chugai.

## Authors’ contributions

JW contributed to the conception and design of the study, data collection and analysis, manuscript writing and final approval of the manuscript. G-RB, HS, AK, CR, H-PT, AR-R, PB-B and SW contributed to data collection and analysis, critical revision and final approval of the manuscript. IHR contributed to the conception and design of the study, financial support, manuscript writing, and final approval of the manuscript. TD contributed to data collection and analysis, manuscript writing and final approval of the manuscript. All authors read and approved the final version of the manuscript.

## Supplementary Material

Additional file 1 **List of study investigators and sites.** Alphabetical list of all study investigators and their locations. Click here for file

## References

[B1] Edwards JC Szczepanski L Szechinski J Filipowicz-Sosnowska A Emery P Close DR Stevens RM Shaw T Efficacy of B-cell-targeted therapy with rituximab in patients with rheumatoid arthritis N Engl J Med 2004 350 2572 2581 10.1056/NEJMoa032534 15201414

[B2] Keystone E Fleischmann R Emery P Furst DE van Vollenhoven R Bathon J Dougados M Baldassare A Ferraccioli G Chubick A Udell J Cravets MW Agarwal S Cooper S Magrini F Safety and efficacy of additional courses of rituximab in patients with active rheumatoid arthritis: an open-label extension analysis Arthritis Rheum 2007 56 3896 3908 10.1002/art.23059 18050221

[B3] Buch MH Smolen JS Betteridge N Breedveld FC Burmester G Dörner T Ferraccioli G Gottenberg JE Isaacs J Kvien TK Mariette X Martin-Mola E Pavelka K Tak PP van der Heijde D van Vollenhoven RF Emery P Rituximab Consensus Expert Committee Updated consensus statement on the use of rituximab in patients with rheumatoid arthritis Ann Rheum Dis 2011 70 909 920 10.1136/ard.2010.144998 21378402 PMC3086093

[B4] Keystone EC Cohen SB Emery P Kremer JM Dougados M Loveless JE Chung C Wong P Lehane PB Tyrrell H Multiple courses of rituximab produce sustained clinical and radiographic efficacy and safety in patients with rheumatoid arthritis and an inadequate response to 1 or more tumor necrosis factor inhibitors: 5-year data from the REFLEX study J Rheumatol 2012 39 2238 2246 10.3899/jrheum.120573 23027887

[B5] van Vollenhoven RF Emery P Bingham CO Keystone EC Fleischmann RM Furst DE Tyson N Collinson N Lehane PB Long-term safety of rituximab in rheumatoid arthritis: 9.5-year follow-up of the global clinical trial programme with focus on adverse events of interest in RA patients Ann Rheum Dis 2013 72 1496 1502 10.1136/annrheumdis-2012-201956 23136242 PMC3756452

[B6] Zink A Strangfeld A Schneider M Herzer P Hierse F Stoyanova-Scholz M Wassenberg S Kapelle A Listing J Effectiveness of tumor necrosis factor inhibitors in rheumatoid arthritis in an observational cohort study: comparison of patients according to their eligibility for major randomized clinical trials Arthritis Rheum 2006 54 3399 3407 10.1002/art.22193 17075823

[B7] Chatzidionysiou K Lie E Nasonov E Lukina G Hetland ML Tarp U Gabay C van Riel PL Nordström DC Gomez-Reino J Pavelka K Tomsic M Kvien TK van Vollenhoven RF Highest clinical effectiveness of rituximab in autoantibody-positive patients with rheumatoid arthritis and in those for whom no more than one previous TNF antagonist has failed: pooled data from 10 European registries Ann Rheum Dis 2011 70 1575 1580 10.1136/ard.2010.148759 21571731

[B8] Gottenberg JE Ravaud P Bardin T Cacoub P Cantagrel A Combe B Dougados M Flipo RM Godeau B Guillevin L Le Lot X Haculla E Schaeverbeke T Sibilia J Pane I Abbe A Baron G Mariette X French Society of Rheumatology Prospective follow-up of RA patients (3680 patient/year) treated with rituximab in real life: results from the AIR registry [abstract] Ann Rheum Dis 2011 70 465

[B9] Strangfeld A Eveslage M Kekow J Grabetaler A Kaufmann J Listing J Zink A Effectiveness of treatment with rituximab depends on autoantibody status - results from 2 years of experience in the German biologics register RABBIT [abstract] Arthritis Rheum 2009 60 S634 S635

[B10] Vander Cruyssen B Durez P Westhovens R Kaiser MJ Hoffman I De Keyser F MIRA Study Group The Belgian MIRA (MabThera In Rheumatoid Arthritis) registry: clues for the optimization of rituximab treatment strategies Arthritis Res Ther 2010 12 R169 10.1186/ar3129 20831776 PMC2990996

[B11] Van Vollenhoven RF Chatzidionysiou K Nasonov E Lukina G Hetland ML Tarp U Gabay C Van Riel PL Nordstrom DC Gomez-Reino JJ Pavelka K Tomsic M Lie E Kvien TK Six-month results from the collaborative European registries for rituximab in rheumatoid arthritis (CERERRA). Efficacy of rituximab is highest in RF-positive patients and in those who failed at most one prior anti-TNF [abstract]. The 2009 ACR/ARHP Annual Scientific MeetingPhiladelphia, October 16-21, 2009 Arthritis Rheum 2009 60 suppl S624 S625

[B12] Cleveland WS Robust locally weighted regression and smoothing scatterplots J Am Stat Assoc 1979 74 829 836 10.1080/01621459.1979.10481038

[B13] Keystone EC Fleischmann RM Emery P Dougados M Williams S Linnik MD Reynard M Multiple courses of rituximab produce sustained efficacy in patients with rheumatoid arthritis with an inadequate response to one or more TNF Inhibitors [abstract] Arthritis Rheum 2010 62 suppl S133

[B14] Smolen JS Aletaha D Bijlsma JW Breedveld FC Boumpas D Burmester G Combe B Cutolo M de Wit M Dougados M Emery P Gibofsky A Gomez-Reino JJ Haraoui B Kalden J Keystone EC Kvien TK McInnes I Martin-Mola E Montecucco C Schoels M van der Heijde D T2T Expert Committee Treating rheumatoid arthritis to target: recommendations of an international task force Ann Rheum Dis 2010 69 631 637 10.1136/ard.2009.123919 20215140 PMC3015099

[B15] Smolen JS Landewé R Breedveld FC Dougados M Emery P Gaujoux-Viala C Gorter S Knevel R Nam J Schoels M Aletaha D Buch M Gossec L Huizinga T Bijlsma JW Burmester G Combe B Cutolo M Gabay C Gomez-Reino J Kouloumas M Kvien TK Martin-Mola E McInnes I Pavelka K van Riel P Scholte M Scott DL Sokka T Valesini G EULAR recommendations for the management of rheumatoid arthritis with synthetic and biological disease-modifying antirheumatic drugs Ann Rheum Dis 2010 69 964 975 10.1136/ard.2009.126532 20444750 PMC2935329

[B16] MabThera® Summary of product characteristics http://www.ema.europa.eu/ema/index.jsp?curl=pages/medicines/human/medicines/000165/human_med_000897.jsp&mid=WC0b01ac058001d124

[B17] Wendler J Sørensen H Tony H Richter C Krause A Rubbert-Roth A Bogdan M Burmester G Effectiveness and safety of rituximab (RTX) monotherapy compared to RTX combination therapy with methotrexate or leflunomide in the German RTX-treatment of active rheumatoid arthritis in daily practice trial [abstract] Ann Rheum Dis 2009 68 76

[B18] Solau-Gervais E Prudhomme C Philippe P Duhamel A Dupont-Créteur C Legrand JL Houvenagel E Flipo RM Efficacy of rituximab in the treatment of rheumatoid arthritis. Influence of serologic status, coprescription of methotrexate and prior TNF-alpha inhibitors exposure Joint Bone Spine 2012 79 281 284 10.1016/j.jbspin.2011.05.002 21724444

[B19] Owczarczyk K Hellmann M Fliedner G Röhrs T Maizus K Passon D Hallek M Rubbert A Clinical outcome and B cell depletion in patients with rheumatoid arthritis receiving rituximab monotherapy in comparison with patients receiving concomitant methotrexate Ann Rheum Dis 2008 67 1648 1650 10.1136/ard.2007.087023 18854518

[B20] Chatzidionysiou K Lie E Nasonov E Lukina G Hetland ML Tarp U van Riel PL Nordström DC Gomez-Reino J Pavelka K Tomsic M Kvien TK van Vollenhoven RF Gabay C Effectiveness of disease-modifying antirheumatic drug co-therapy with methotrexate and leflunomide in rituximab-treated rheumatoid arthritis patients: results of a 1-year follow-up study from the CERERRA collaboration Ann Rheum Dis 2012 71 374 377 10.1136/annrheumdis-2011-200003 21972242

[B21] Emery P Dörner T Optimising treatment in rheumatoid arthritis: a review of potential biological markers of response Ann Rheum Dis 2011 70 2063 2070 10.1136/ard.2010.148015 22039166

[B22] Isaacs JD Cohen SB Emery P Tak PP Wang J Lei G Williams S Lal P Read SJ Effect of baseline rheumatoid factor and anticitrullinated peptide antibody serotype on rituximab clinical response: a meta-analysis Ann Rheum Dis 2013 72 329 336 10.1136/annrheumdis-2011-201117 22689315

[B23] van Vollenhoven RF Emery P Bingham CO Keystone EC Fleischmann R Furst DE Macey K Sweetser M Kelman A Rao R Long-term safety of patients receiving rituximab in rheumatoid arthritis clinical trials J Rheum 2010 37 558 567 10.3899/jrheum.090856 20110520

