# Supplementary material for: Rituximab in patients with rheumatoid arthritis in routine practice (GERINIS): six-year results from a prospective, multicentre, non-interventional study in 2,484 patients
Source: Arthritis Res Ther. 2014 Mar 26;16(2):R80. doi: 10.1186/ar4521 (PMC4060207; doi:10.1186/ar4521)
Supplement: Additional file 1 — List of study investigators and sites. Alphabetical list of all study investigators and their locations. [file ar4521-S1.pdf]

Additional file 1

List of study investigators and sites

Ben Abendroth, Jena
Mohammed Al-Azem, Lohne
Sebastian Albert, Offenburg
Karl Alliger, Zwiesel
Bianca Andermann, RPB Rheuma Projekt Berlin Brandenburg, Berlin
Peer M. Aries, Hamburg-Altona
Christoph Baerwald, Universität Leipzig Medizinische Fakultät, Leipzig
Sabine Balzer, Bautzen
Erich Bärlin, Ludwigsburg
Peter Bartz-Bazzanella, Klinik für Internistische Rheumatologie, Würselen
Christine Baumann, Plauen
Detlef Becker-Capeller, Stade
Wulf-Dieter Behnert, Kassel
Frank Behrens, Klinikum der Johann Wolfgang Goethe Universität, Frankfurt am Main
Walter Behringer, Herz-Jesu-Krankenhaus, Fulda
Michaela Bellm, Bruchsal
Sylvia Berger, Naunhof
Raoul Bergner, Klinikum der Stadt Ludwigshafen, Ludwigshafen
Michaela Berndt, Dietrich-Bonhoeffer-Klinikum, Neubrandenburg
Werner A. Biewer, Saarbrücken
Stephanie Böddeker, Marl
Stefan Boecker, Frankfurt
Martin Bohl-Bühler, Potsdam
Wilfried Bott, Soltau
Anke Böttger, Landau/Pfalz
Axel Brand, Schlangenbad
Jürgen Braun, Rheumazentrum Ruhrgebiet, St. Josefs-Krankenhaus, Herne
Elke Bräunig, Kahla
Reinhard Bruhn, MEDIAN KLINIK Bad Sülze, Bad Sülze
Gerd-Rüdiger Burmester, Charité Campus Mitte, Berlin
Ewa Chwistek, RPB Rheuma Projekt Berlin Brandenburg, Berlin
Ina Dahmann, Göttingen
Winfried Demary, Hildesheim
Rainer Dockhorn, Weener
Ines Dornacher, Heidelberg
Elke Drexler, Mönchengladbach
Roman Eder, Deggendorf
Andreas Engel, Stuttgart
Jörg Engels, Koblenz
Thomas Feist, Halle

Eleonore Fiedler, Nordhausen
Christoph Fiehn, Rheumazentrum Baden-Baden GmbH, Baden-Baden
Paul Flaxenberg, Essen
Karin Fleßa, Hof/Saale
Gerhard Fliedner, Osnabrück
Ivan Foeldvari, Hamburg
Johannes Frey, Neuburg a. d. Donau
Henry Fricke-Wagner, Zwickau
Petra Fuchs, Augsburg
Arne Gäfgen, Knappschaftskrankenhaus Bottrop, Bottrop
Ino K. Gao, Heidelberg
Georg Gauler, Osnabrück
Angela Gause, Hamburg
Thomas Geiler, Klinik an der Weißenburg, Uhlstädt-Kirchhasel
Lars Gerlach, Niefern-Oeschelbronn
Andreas Göbel, Lippstadt
Karl-Heinz Göttl, Passau
Anett Gräßler, Pirna
Conny Ullrich Günther, Krankenhaus Waltershausen Friedrichroda GmbH, Friedrichroda
Judith Günzel, Dortmund
Irmgard Gürtler, Neuss
Bernd Häckel, Frankenberg
Kora Hahn, Bezirksklinikum Obermain, Ebensfeld
Frank Hamann, Leipzig
Johannes Häntsch, Darmstadt
Peter Härle, St. Vincenz-und-Elisabeth-Hospital, Mainz
Werner Harmuth, Marktredwitz
Roland Haux, Berlin
Reinhard Hein, Nienburg
Carl-Johannes Heinmüller, Wuppertal
Stefan Heitmann, Marienhospital Stuttgart, Stuttgart
Martin Hesse, Bad Kreuznach
Guido Hoese, Stadthagen
Maria Höhle, Hamburg
Ann-Dörthe Holst, Ludwigslust
Peter Hrdlicka, Zeisigwaldkliniken Bethanien, Chemnitz
Georg Hübner, Lingen
Steffen Illies, Schwerte
Swen H. Jacki, Tübingen
Wolf-Oliver Jordan, Lehrte
Ulf Kaeding, Parchim
Irmingard Kamilli, München
Andreas Kapelle, Hoyerswerda
Kirsten Karberg, Berlin
Thomas Karger, Köln
Ulrich Käßer, Gießen

Peter Kästner, Erfurt
Jörg Kaufmann, Ludwigsfelde
Herbert Kellner, München
Gernot Keyßer, Martin-Luther-Universität Halle-Wittenberg, Halle/Saale
Hans-Peter Klein, Neustadt
Thilo Klopsch, Neubrandenburg
Michael Koch, Erfurt
Ingrid Kölnberger, Bogen
Gernot Kratzsch, Ulm
Holger Krauel, Asklepios Klinik f. Innere Medizin, Weißenfels
Dietmar Krause, Gladbeck
Jens-Olaf Krause, Regensburg
Lutz Kriegel, Nürnberg
Eveline Krieger-Dippel, München
Brigitte Krummel-Lorenz, Frankfurt
Cornelia Kühne, Haldensleben
Heinz-Jürgen Lakomek, Johannes Wesling Klinikum Minden, Minden
Hans-Eckhard Langer, Düsseldorf
Andreas Lauffer, Karlsruhe
Karin Leumann, Riesa
Anke Liebhaber, Halle/Saale
Thomas Linde, Halle/Saale
Gabriele Lorenz, Chemnitz
Hanns-Martin Lorenz, Uniklinik Heidelberg, Heidelberg
Kirsten Lüthke, Dresden
Ulrich Maaß, Güstrow
Jozo Majdandzic, Mainz
Anja Maltzahn, Kassel
Helga Manschwetus, Braunschweig
Thomas Marycz, Amberg
Johannes Mattar, Überlingen
Ursula Mauß-Etzler, Karlsruhe
Lothar Mayer, Bad Staffelstein
Werner-Johannes Mayet, Nordwest-Krankenhaus Sanderbusch, Sande
Lothar Meier, Hofheim
Adelheid Melzer, Seesen
Hans-Jürgen Menne, Dortmund
Eckart Möbius, Schwerin
Harald Mörtlbauer, Städt. Klinikum München GmbH, München
Michael Müller, Freiberg
Gerhard-Anton Müller, Georg-August-Universität, Göttingen
Ulf Müller-Ladner, Kerckhoff-Klinik Forschungsgesell.GmbH, Bad Nauheim
Alexander Natusch, Berlin*
Gunther Neeck, Biomedro, Bad Doberan
Joachim Neuwirth, Norderstedt
Gabriele Niederbiermann-Koczy, Steinhagen

Elisabeth Niewersch, Marburg
Mathias Nitsch, Waldkrankenhaus Rudolf Elle, Eisenberg
Ernst Nitsche, Regensburg
Hubert Nüßlein, Nürnberg
Wolfgang Ochs, Bayreuth
Matthias Oelsner, Gera
Peter Oelzner, FSU Klinik f. Innere Medizin III, Jena
Artur Operhalski, Kreisklinik Aschersleben/Staßfurt, Aschersleben
Dorothea Pick, Bad Neuenahr
Manfred Piegsa, Gießen
Andreas Reck, Mittelherwigsdorf
Sven Remstedt, Berlin
Matthias Richter, Rostock
Constanze Richter, Stuttgart
Beate Roch, Dresden
Karin Rockwitz, Goslar
Bernhard Rößner, Erfurt
Ekkehard Röther, Donaueschingen
Andrea Rubbert-Roth, Uniklinik Köln, Köln
Herwig Rumpel, Regensburg
Gernot Scheibl, Paderborn
Georg Schett, Universitätsklinikum Erlangen, Erlangen
Heinz Schleenbecker, Rheumazentrum Mittelhessen GmbH & KG, Bad Endbach
Thomas Schleiffer, Wilhelmshaven
Maria-Anna Schleußner, Heilbad Heiligenstadt
Albert Schmid, Bielefeld
Hagen Schmidt, Berlin
Reinhold-Ernst Schmidt, Med. Hochschule Hannover, Hannover
Matthias Schneider, Universitätsklinikum Düsseldorf, Düsseldorf
Ulrich Schoo, Rheine
Andreas Schramm, Freiburg
Agnes-Lotte Schrepler-Konya, Mannheim
Frank Schumann, Reken
Andreas Schwarting, Uniklinik Mainz, Mainz
Ilka Schwarze, Leipzig
Cornelia Schweder, Hagen
Reiner Schwenke, Dresden
Gudrun Schwenke, Johanniter Krankenhaus, Treuenbrietzen
Carola Schwerdt, Dessau
Angela Seifert, Immanuel Krankenhaus GmbH, Berlin
Marco Semmler, Klinikum Südstadt, Rostock
Helmut Sörensen, RPB Rheuma Projekt Berlin Brandenburg, Berlin
Christof Specker, Kliniken Essen-Süd, Essen
Wolfgang Spieler, Zerbst
Marie-Luise Stadelmann, Wittlich
Elisabeth Ständer, Schwerin

Carsten Stille, Hannover
Frank Striesow, Bonn
Harald Strothmeyer, Düsseldorf
Johannes Strunk, Krankenhaus Köln - Porz, Köln
Martin Talke, Berlin
Andreas Teipel, Leverkusen
Astrid Thiele, St. Josefs-Zentrum für Orthopädie u. Rheumatologie, Wuppertal
Wolfgang Thies, Herrsching
Hans-Peter Tony, Medizinische Klinik und Poliklinik II, Würzburg
Andreas Trabandt, Kreiskrankenhaus Demmin, Demmin
Frank Trautmann, Mainz
Harald Tremel, Hamburg
Igor Turin, Karlstadt
Paul Veress, Mönchengladbach
Reiner Vogt, Petershagen
Andreas Völker, Ahlen
Markus Vollmer, Mönchengladbach
Ulrich von Hinüber, Hildesheim
Hubertus von Wilmowsky, Knappschaftskrankenhaus, Püttlingen
Jan Voswinkel, Universitätsklinikum des Saarlandes, Homburg
Peter Wagener, Nienburg
Jürgen Währisch, Essen
Jochen Walter, Rendsburg
Uta Walter, Salzwedel
Siegfried Wassenberg, Ratingen
Sven Weidner, Stuttgart
Martin Welcker, Planegg
Jörg Wendler, Erlangen
Barbara Wenzel, Immanuel Krankenhaus GmbH, Berlin
Maria Westerhoff, Rheinfelden
Jens Westphal, Schramberg-Sulgen
Elke Wilden, Köln
Peter Willeke, Kooperatives Rheumazentrum Münster e. V., Münster
Stefan Wolf, Sinsheim
Wolf-Dieter Wörth, Wiesbaden
Michael Zänker, Rheumazentrum Nord-Brandenburg e.V., Bernau
Silke Zinke, Berlin

[*]Deceased.
